# An Innovative in Situ Monitoring of Sulfate Reduction within a Wastewater Biofilm by H_2_S and SO_4_^2−^ Microsensors

**DOI:** 10.3390/ijerph17062023

**Published:** 2020-03-19

**Authors:** Hong Liu, Xun Liu, Ning Ding

**Affiliations:** 1 School of Environmental Science and Engineering, Jiangsu Key Laboratory of Environmental Science and Technology, Suzhou University of Science and Technology, Suzhou 215009, China; 2 School of Civil Engineering, Suzhou University of Science and Technology, Suzhou 215000, China; liuxun8127@163.com; 3 Key Laboratory of Cleaner Production and Comprehensive Utilization of Resources, China National Light Industry, Department of Environmental Science and Engineering, Beijing Technology and Business University, Beijing 100048, China; dingning@btbu.edu.cn

**Keywords:** microelectrode, wastewater, biofilm, sulfate reduction, activity

## Abstract

Microelectrodes can be used to obtain chemical profiles within biofilm microenvironments. For example, sulfate (SO_4_^2−^) and hydrogen sulfide (H_2_S) microelectrodes can be used to study sulfate reduction activity in this context. However, there is no SO_4_^2−^ microelectrode available for studying sulfate reduction in biofilms. In this study, SO_4_^2−^ and H_2_S microelectrodes were fabricated and applied in the measurement of a wastewater membrane-aerated biofilm (MAB) to investigate the in situ sulfate reduction activity. Both the SO_4_^2−^ and H_2_S microelectrodes with a tip diameter of around 20 micrometers were successfully developed and displayed satisfying selectivity to SO_4_^2^^−^ and H_2_S, respectively. The Nernstian slopes of calibration curves of the fabricated SO_4_^2−^ electrodes were close to −28.1 mV/decade, and the R^2^ values were greater than 98%. Within the selected concentration range from 10^−^^5^ M (0.96 mg/L) to 10^−^^2^ M (960 mg/L), the response of the SO_4_^2−^ microelectrode was log-linearly related to its concentration. The successfully fabricated SO_4_^2−^ microelectrode was combined with the existing H_2_S microelectrode and applied on an environmental wastewater biofilm sample to investigate the sulfate reduction activity within it. The H_2_S and SO_4_^2−^ microelectrodes showed stable responses and good performance, and the decrease of SO_4_^2−^ with an accompanying increased of H_2_S within the biofilm indicated the in situ sulfate reduction activity. The application of combined SO_4_^2−^ and H_2_S microelectrodes in wastewater biofilms could amend the current understanding of sulfate reduction and sulfur oxidation within environmental biofilms based on only H_2_S microelectrodes.

## 1. Introduction

Biofilms play significant roles in wastewater treatment due to their environmental friendliness, cost effectiveness, and strong adaptability to wastewater quality as compared to other biological treatments. Multispecies biofilms contain a high cell density and reflect complex metabolic activity [1,2]; therefore, their potential for in situ bioremediation of wastewater has been investigated. There have been studies focused on biofilm reactors that have proven useful for treating various wastewaters [3,4,5,6]. Previous studies have demonstrated the heterogeneous structure of wastewater biofilms [7]. There could be combined microbial processes within a single piece of biofilm with stratification, which could simultaneously include aerobic oxidation, nitrification, denitrification, and sulfate reduction [8,9]. Of these, sulfate reduction is an important microbial process, since various kinds of wastewater contain high sulfate concentrations. Sulfate is converted to sulfide, which can combine with hydrogen to form hydrogen sulfide (H_2_S) or combine with metal to form metal sulfide precipitation. If water containing sulfate is not treated properly, H_2_S or metal sulfide produced by the sulfate reduction process might have a negative impact on the environment. Thus, studying sulfate reduction process in biofilm microenvironments is of great importance to better understand the performance of biofilm reactors.

Microelectrodes have proved to be effective tools for determining in situ microbial activity by obtaining chemical concentration gradients in biofilm microenvironments [10]. Microelectrodes such as oxygen [11], H_2_S [12], hydrogen [13], nitrous oxide [14], pH [15], ammonium [16], nitrate [17], nitrite [18], and sulfide [19] have been successfully developed and calibrated for profiling chemical concentrations in biofilm microenvironments. Microelectrodes have also been successfully applied in the study of sulfate reduction processes in biofilm, (e.g., sulfide dynamics in aerobic biofilms were investigated using sulfide microelectrodes combined with oxygen and pH microelectrodes [20]). Kuhl et al. designed an H_2_S microelectrode and detected sulfate reduction activity in the anoxic zone of the biofilm [21]. Yu and Bishop [22] conducted research on the oxidation-reduction potential (ORP) change in a wastewater biofilm using O_2_ and S^2−^ microsensors; it was also found that sulfate reduction took place in a deeper section of the biofim. Ramsing et al. [23] first addressed combining microelectrodes and molecular analysis to study sulfate-reducing bacteria (SRB) in a wastewater biofilm. Results found that less SRB cells were present in the biofilm while the oxygen concentration was high. Okabe et al. [24] studied the vertical distribution and in situ activity of SRB in wastewater biofilm from microbial and chemical persipecives based on combined microelectrodes and molecular analysis. Results showed that SRB activity can be only found in a narrow zone (150~300 µm) within biofilm. Liu et al. [25] studied the vertical distribution of SRB and its activity with a wastewater biofilm based on H_2_S microelectodes. Ito et al. [26] also proved that an increase of SRB cells results in a more active sulfide production in a wastewater biofilm.

Although much research have been conducted on sulfate reduction activities, most of the studies focusing on the internal sulfur cycle in biofilms have to our knowledge been based on H_2_S microelectrodes, due to the lack of sulfate microelectrodes with which to directly determine sulfate in biofilm microenvironments. Therefore, the objective of the present study was to develop H_2_S and SO_4_^2−^ microelectrodes to evaluate their applicability in wastewater biofilm microenvironments; the obtained chemical profiles of H_2_S and SO_4_^2−^ within wastewater biofilm based on H_2_S and SO_4_^2−^ microelectrodes were used to indicate sulfate reduction activity. The present study will provide comprehensive information to improve our current understanding of the sulfate reduction process in biofilm microenvironments.

## 2. Material and Methods

### 2.1. Set Up of Biofilm Reactor

**Figure 1:**
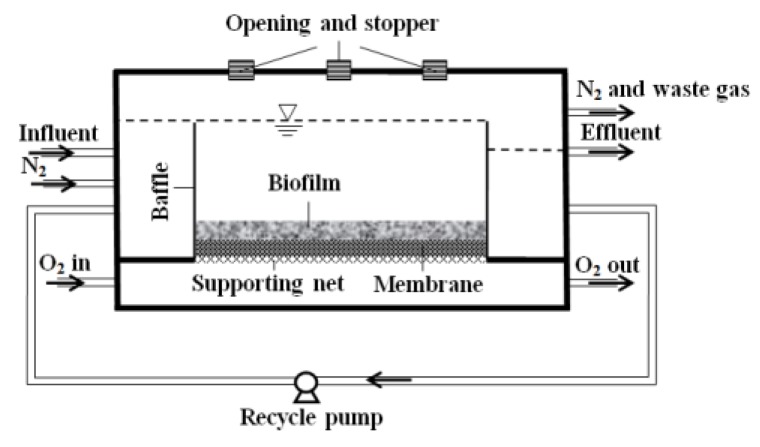
Setup of the membrane-aerated biofilm reactor (MABR).

An innovative membrane-aerated biofilm reactor (MABR) was developed to culture biofilm for microelectrode measurement, as shown in Figure 1. The openings on the cover of the reactor were sealed by stoppers, and could be removed during microelectrode measurement. The total volume of the reactor was 0.9 L. The substratum selected for biofilm attachment and growth were dense silicone flat-sheet membranes (Model: SSP-M823, Specialty Silicone Products, Inc., pore size: 0.12 × 0.04 μm, 25 μm thick, polypropylene; Celgard, NC, USA).

At the initial stage of biofilm culture, activated sludge collected from the anaerobic digester at the Gold Bar Wastewater Treatment Plant in Edmonton was inoculated into the reactor. After successfully starting up, pure O_2_ with a flowrate of 20 mL/min was flowed through the silicone membrane. N_2_ was purged into the influent to make the synthetic wastewater O_2_-free.

The MABR was operated for more than a year. Bulk reactor parameters were monitored every two to three days. Dissolved oxygen (DO) concentration, pH, and ORP were measured in the influent and effluent. Oxygen membrane electrodes (Model: Orion 97-08, Thermo Electron Corporation) with a DO meter (Model: 50B, YSI Inc., Yellow Springs, OH, USA) were used for DO measurement. pH electrodes (Cat.# 13-620-108, Accumet, Fisher Scientific, Edmonton, Canada) with a pH meter (AR 15, Accumet, Fisher Scientific) were used for pH measurement. ORP electrodes (Cat.# 13-620-81, Accumet, Fisher Scientific) with a pH meter (AR 25, Fisher Scientific) were used for ORP measurements. Chemical Oxidation Demand (COD) was analyzed using a Digital Reactor Block 200 digester (Model: DRB 200, Hatch). For SO_4_^2−^ determination, the influent and effluent samples were filtered with 0.45-μm membrane filters before analysis using ion chromatography (Model: ICS-2000, Dionex).

The synthetic wastewater was composed of 250 mg/L dextrose (COD), 5 mg/L KH_2_PO_4_, 20 mg/L NH_4_Cl, 277.5 mg/L Na_2_SO_4_, 12.86 mg/L MgCl_2_·6H_2_O, 2.57 mg/L FeSO_4_·7H_2_O, 0.26 mg/L CoCl_2_·6H_2_O, 0.77 mg/L CaCl_2_·2H_2_O, 0.26 mg/L CuSO_4_·H_2_O, 0.26 mg/L MnCl_2_·4H_2_O, 0.26 mg/L ZnSO_4_·7H_2_O, and 1 mg/L yeast extract. The influent rate of wastewater was set as 2.0 mL/min. The reactor was operated at room temperature and at pH 7.6 ± 0.2, with a recirculation rate of around 200 mL/min.

### 2.2. Microelectrodes Preparation

#### 2.2.1. H_2_S Microelectrode

The H_2_S microelectrodes contained a working anode, guard anode, and reference cathode. The detailed fabrication procedures of the combined H_2_S microelectrode are as follows: the working anode was prepared by cutting a platinum wire (50 µm diameter) into five sections. The wire was cleaned with Deionized (DI) water. The tip of the platinum wire was etched to 5–10 µm in 1 M alkaline (pH > 13) Potassium Cyanide (KCN) solution in a well-ventilated fume hood by vertically dipping in and out until it reached the expected tip size; usually, the smaller the tip size, the best performance it had. The etched platinum wire was cleaned by immersing it sequentially in three beakers of Milli-Q water, after which the platinum wire was inserted into the micropipette. Next, the pipette was hung in the center of a trough heating filament (Sutter Instruments) to taper the tip capillary. The tip (Pt wire coated with glass) was ground down using a micropipette grinder to exposure the wire. The working anode and working cathode Ag/AgCl were inserted into a tapered outer casing. The distance between the tip of the working anode and the outer casing was about 30–40 μm. The distance between the tip of the working anode and the guard anode was about 150–300 μm.

#### 2.2.2. SO_4_^2−^ Microelectrode

The SO_4_^2−^ microelectrode applied in this study belonged to a liquid ion selective microelectrode. The fabrication procedures were as follows: glass micropipettes were pulled firstly in a vertical pipette puller (PUL-100, World Precision Instruments Inc., Sarasota, FL, USA). Then, the tips of the pulled micropipettes were broken by fine tweezers under a microscope (Stemi SV11, Carl Zeiss, Canton, MA, USA). The micropipette tips were dipped into salinization reagent N, N-dimethyltrimethylsilylamine (Cat.# 41716, Fluka, Ronkonkoma, New York, NY, USA) for 5 s then put into an oven at 180 °C for 24 h. After salinization, the micropipette tip was immersed into membrane solution, and the reference electrolyte was filled into the micropipette at the other side of the micropipette. The membrane components of sulfate microelectrodes are shown in Table 1.

### 2.3. Microelectrodes Calibration

#### 2.3.1. Calibration of H_2_S Microelectrodes

**Figure 2:**
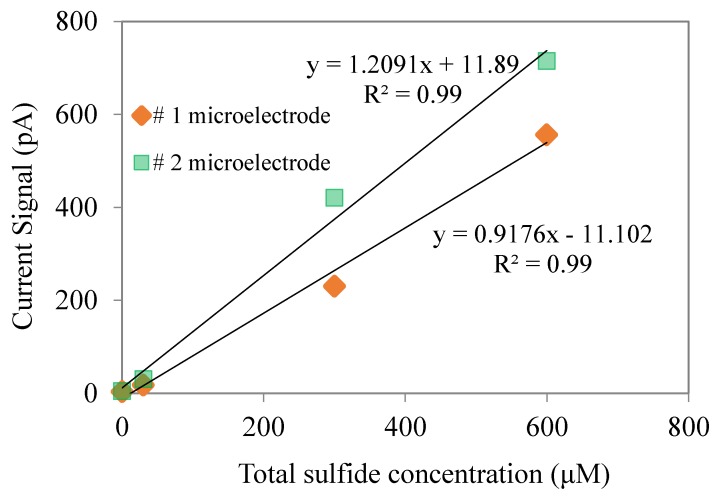
Calibration curve of H_2_S microelectrodes.

The response of H_2_S microsensors could be related to pH values. In this study, we calibrated H_2_S microelectrodes under neutral pH conditions, as our wastewater biofilm was operated under neutral pH conditions. Standard solutions of H_2_S (0–400 M) were prepared by transferring certain amounts of the Na_2_S stock into the de-aerated pH 7.0 phosphate buffer. The calibration curve is shown in Figure 2. It can be seen that the H_2_S are linearly related to the current signal with a R2 higher than 0.99.

#### 2.3.2. Calibration of SO_4_^2−^ Microelectrodes

**Figure 3:**
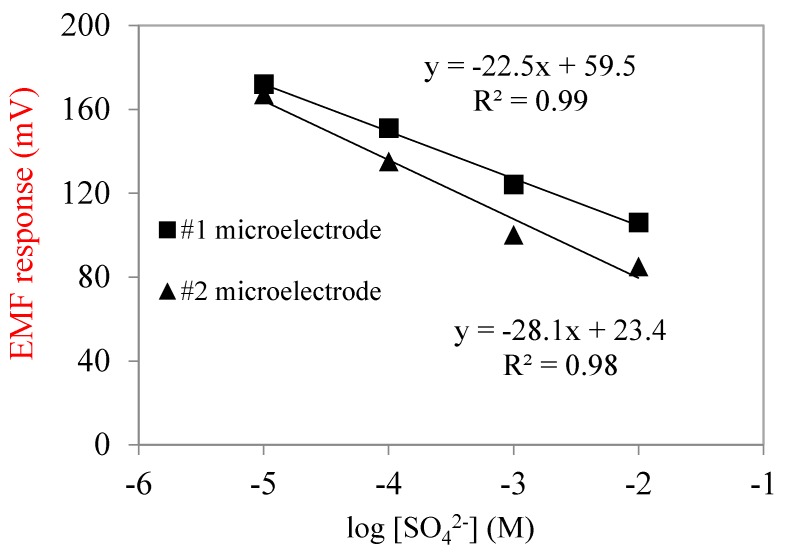
Calibration curve of SO_4_^2−^ microelectrodes.

The fabricated SO_4_^2−^ microelectrodes needed to be calibrated and validated before measurement. The linear relationship between electromotive force (EMF) and sulfate concentration was used to indicate microelectrode performance. The calibration curve is shown in Figure 3. In this study, the Nernstian slopes of calibration curves were close to −28.1 mV/decade, and the R^2^ values were greater than 98%. Within the selected concentration range from 10^−5^ M (0.96 mg/L) to 10^−2^ M (960 mg/L), the response of sulfate microelectrodes was log-linearly related to their concentration. Sulfate concentrations in domestic wastewater are usually from 20 to 500 mg/L; therefore, the sulfate microelectrodes fabricated in this study covered the concentration detection range.

#### 2.3.3. QA/QC of H_2_S and SO_4_^2−^ Microelectrodes

Each microsensor had to be calibrated before and after measurement, because during measurement, the sensor tip might be broken, or there might be particles in bulk water or within biofilm that could adsorb on the surface of the sensor tip, and thus affect the microsensor performance. Calibration before and after measurement ensures the microsensors are working well. Usually, we fabricated several microsensors at one time, with each one was calibrated before and after measurement. If calibration was not working after measurement, then another calibrated microsensor was used to repeat the measurement.

### 2.4. Net Consumption and Production Rates Calculation

Net consumption and production rates of H_2_S and SO_4_^2−^ were estimated from the H_2_S and SO_4_^2−^ concentration profiles based on Fick’s second law of diffusion [27], ∂*C*(*z*,*t*)/∂*t* = *D* × ∂^2^*C*(*z*,*t*)/∂*z*^2^ − *R*(*z*) *+ P(z)*, where *C*(*z*,*t*) is the concentration at time *t* and depth *z*, *D* is the molecular diffusion coefficient in the liquid phase, *R* is the net specific consumption rate, and *P* is the net production rate. Based on the H_2_S concentration profile, using Fick’s first law of diffusion *J* = −*D*(d*C*(*z*,*t*)/d*z*), the flux *J* was obtained. The activity profile was derived from these two equations. The values 1.39 × 10^−5^ cm^2^/s and 0.6× 10^−5^ cm^2^/s were used for the sulfide and sulfate diffusion coefficients, respectively [25].

## 3. Results and Discussions

### 3.1. Reactor Performance

The MABR was inoculated with activated sludge to initiate the biofilm growth. The reactor was operated in batch mode in the first two days with a recycling rate of 100 mL·min^−1^. The reactor was switched to continuous flow mode with synthetic wastewater (composition of the wastewater is shown in Section 2.1) continuously flowed into the reactor. The influent flow rate was maintained at 2 mL·min^−1^, the recirculation rate was set as 200 mL·min^−1^, and the hydraulic retention time was maintained at 5.6 h. The average influent SO_4_^2−^ concentration was measured as 227 mg/L. The SO_4_^2−^ loading in the biofilm reactor was calculated as 0.97 × 10^6^ mg/m^3^ × d. The water characteristics in MABR are shown in Table 2. In the flowing bulk water, the DO concentration was kept less than 0.85 mg/L, ORP was −300 ± 50 mV, and pH 7.6 ± 0.2.

It has been well studied that anaerobic conditions, a proper pH, and a highly reduced environment are necessity for SRB growth [28]. Although SRB can grow and live in extreme pH conditions [29], the optimal pH range is within 5–9 [30]. In the present study, the average SO_4_^2−^ removal efficiency reached up to 62%, indicating that sulfate reduction was occurring in the membrane-aerated biofilm (MAB). Results were consistent with a previous study showing that sulfate reduction could happen within a single piece of biofilm [9,25,31,32].

### 3.2. Microelectrodes Biofilm Measurement

**Figure 4:**
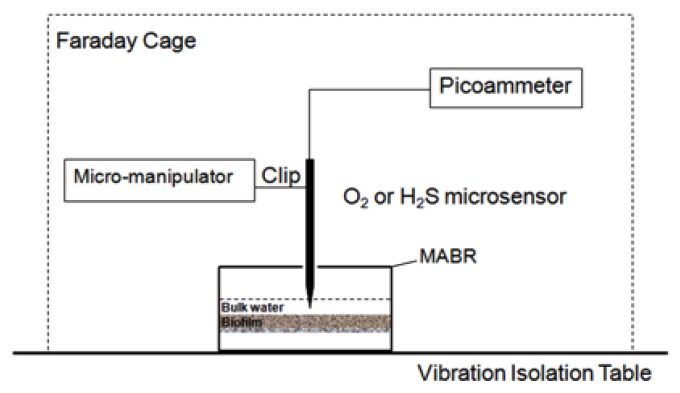
An illustration of the microelectrode measurement setup.

Biofilm thickness was measured before conducting microelectrode measurement. A glass micropipette mounted on a micromanipulator was positioned on the surface of the biofilm, then moved down at a step size of 20 m until it touched the bottom. The movement was controlled by the manipulator and viewed through a microscope. In our case, biofilm thickness was measured as 1000 m. In situ microelectrode measurements of SO_4_^2−^ and H_2_S within the biofilm were conducted. An illustration of the microelectrode measurement setup is shown in Figure 4.

All the measurements were made on a high-performance vibration isolation table (Technical Manufacturing Corporation, USA) in a Faraday cage (Technical Manufacturing Corporation, USA) to avoid external vibration and interference. Right before and after each measurement, microelectrodes needed to be calibrated. During calibration, the microelectrode and reference milli-electrode (Microelectrodes, Inc., USA, MI-401) were both immersed in a standard solution and EMF signals were recorded by a sensitive high-resistance electrometer (Keithley, Cleveland, OH, USA, 6517). During measurements, microelectrodes were mounted on a micromanipulator (Model M3301R, World Precision Instruments, Inc., Sarasota, FL, USA), and the microelectrode tips were advanced into the biofilm from the surface to a deeper section at the specific proper step size, usually 10 to 50 μm. Through a microscope (Model: Stemi SV11, Carl Zeiss, Jena, Germany) it could be seen whether the tip had touched the biofilm surface, and microelectrode movement was controlled through movement of the micromanipulator. The calibration curves of the SO_4_^2−^ microelectrodes are shown in Figure 3, based on the process listed in Table 1. The average calibration curve slopes were −25.3 mV/decade, which is close to the ideal slope of −29.6 mV/decade. The sulfate microelectrodes showed a good selectivity towards sulfate ions, resulting in the present study being consistent with previous research [33].

**Figure 5:**
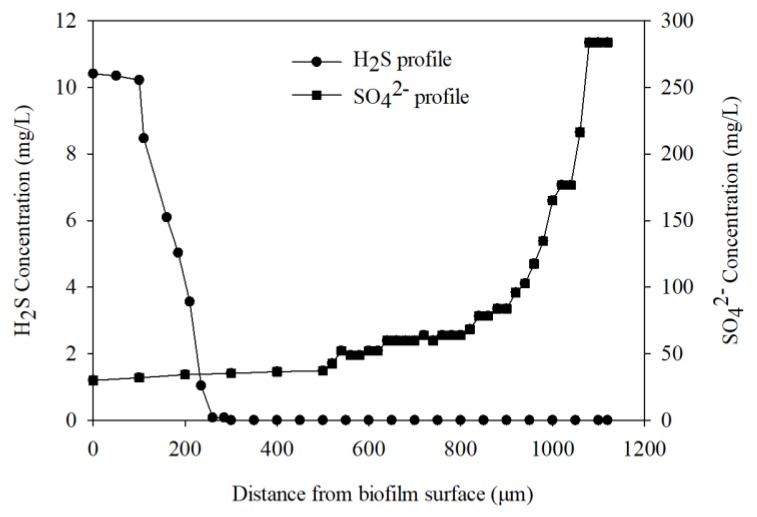
H_2_S and SO_4_^2−^ microelectrode profiles within the biofilm.

As shown in Figure 5, the H_2_S concentration profile showed that H_2_S production was limited to the zone near biofilm surface (0–285 µm, setting 0 as the interface between bulk water and biofilm) of the biofilm, while sulfate gradually decreased from 283 mg/L at the bottom of the biofilm near the membrane surface until it reached about 35 mg/L near the surface biofilm (anoxic zone). The sulfate reduction activity could not only be indicated by the average sulfate removal in bulk water phase, but also by the production of H_2_S along with a decrease of sulfate within the biofilm microenvironment. This expands on previous research that showed sulfate reduction activity to rely solely on sulfide microelectrodes [20].

### 3.3. Estimation of Consumption and Production Rate of H_2_S and SO_4_^2−^

**Figure 6:**
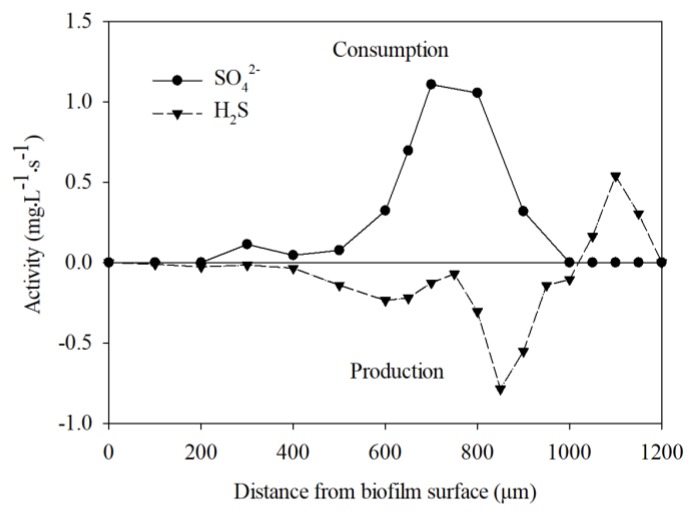
The net specific consumption and production rates of H_2_S and SO_4_^2−^ in the membrane-aerated biofilm (MAB) biofilm.

As shown in Figure 6, high levels of sulfate-reducing activity (H_2_S production rates around 0.80 mg·L^−1^·s^−1^) were found at around 400–1000 μm below the interface. Below the anoxic zone, at about 1000–1200 μm below the interface where oxygen was present, H_2_S was oxidized and consumption was observed. Sulfate consumption of approximately 1.1 mg·L^−1^·s ^−1^ occurred about 600–1000 μm below the biofilm–liquid interface. A previous study established a model to describe sulfate reduction activity in a wastewater biofilm [34], while other recent studies have evaluated the effects of diversified operating strategies and external factors on sulfate reduction activity [35,36]. Most of the current studies on sulfate reduction activity have been based on characteristic parameters in bulk water instead of within biofilm microenvironments. This was the first time that we used combined H_2_S and SO_4_^2−^ microsensors to study sulfate reduction activity within a biofilm microenvironment. Microelectrode measurements for in situ metabolic activity in this study improved on previous studies [24,26] in that the measurements were conducted under actual growth conditions in the MABR. All measurements were performed during operations of the reactor in place of taking biofilm samples out; thus, results reflect the in situ consumption or production of sulfate reduction microbial activity. The consumption of sulfate and production of H_2_S within the biofilm further demonstrate the sulfate reduction activity within the MAB biofilm.

Based on the consumption of H_2_S accompanying the production of SO_4_^2−^ at the surface anoxic zone of the biofilm (400–1000 μm), it is speculated that sulfate-reducing species were present in this region to conduct the sulfate reduction activity. Findings here correspond with previous studies showing that sulfate-reducing species can be present in the anoxic zone within biofilms [9,24]. A detailed molecular analysis of gene information and spatial distribution of sulfate -reducing bacteria will be needed in further research.

**Figure 7:**
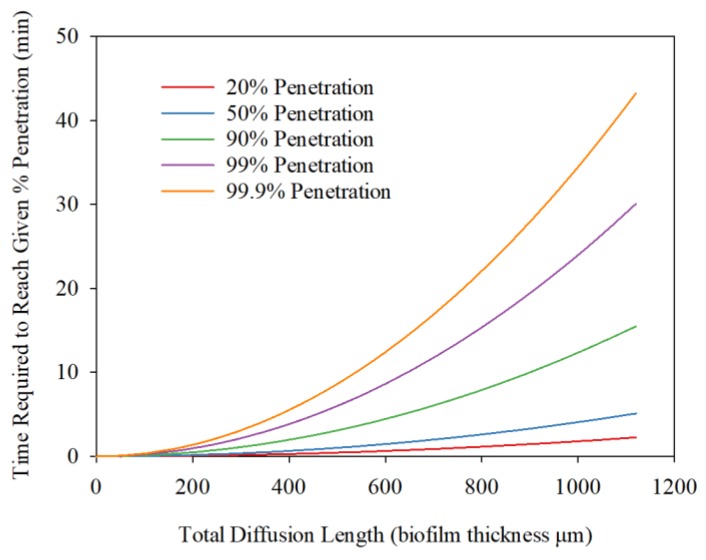
Estimated time required for diffusion out of H_2_S from biofilm.

In order to further investigate the diffusion and reaction of H_2_S within the biofilm, the required diffusion time was estimated based on t = FL^2^/D, where F was obtained from Per Heisler Charts, D is the diffusion coefficient, and L is biofilm thickness. When considering the transport and reaction of chemicals in biofilm, it is necessary to estimate the time required for diffusion through the biofilm by assuming that reactions were not considered, which helps clarify observations when considering diffusions and reactions simultaneously. In the case of diffusion only, the time required to reach 20%, 50%, 90%, 99%, and 99.9% of the H_2_S concentration out of biofilm are shown in Figure 7. For example, 30 min are needed for 99% of the produced H_2_S reach to the biofilm surface, and 5 min will be needed for 50% of the produced H_2_S to be diffused from the biofilm at a 1120-μm thickness.

## 4. Conclusions

H_2_S and SO_4_^2−^ microelectrodes were developed to allow the direct measurement of H_2_S and sulfate, and to indicate sulfate reduction activity within a wastewater biofilm. Specially, sulfate microelectrodes were successfully developed and responded well to sulfate concentration change within a wastewater biofilm microenvironment. The SO_4_^2−^ microelectrode, with a tip diameter of 20 m, exhibited a log-linear response to a sulfate concentration change between 10^−5^ M (1.0 mg L^−1^) and 10^−2^ M (960 mg L^−1^) SO_4_^2−^. The fabricated SO_4_^2−^ microelectrodes could detect sulfate concentrations as low as 10^−5^ M (1.0 mg L^−1^) SO_4_^2−^, with a response time of 90 s. Based on combined H_2_S and SO_4_^2−^ microelectrode measurements, the increasing H_2_S and corresponding decrease in SO_4_^2−^ indicated an in situ microbial sulfate reduction activity within the biofilm. The combination of H_2_S and SO_4_^2−^ microelectrodes is expected to provide a way to directly measure sulfate reduction activity in other microenvironments, which could amend the current research on sulfate reduction activity that relies solely on sulfide microelectrodes. The present study only evaluated the applicability of SO_4_^2−^ microelectrodes in wastewater biofilm, and the application and evaluation of SO_4_^2−^ microelectrodes in other microenvironments (such as sediment) are suggested. The performance optimization of SO_4_^2−^ microelectrodes in other microenvironments needs further exploration.

## Figures and Tables

**Table 1:** Membrane components of the sulfate microelectrodes.

Components	Wt%	Chemical Formula
sulfate ionophore (Fluka 17892)	2% wt	C_22_H_22_N_4_S_2_ (1,3-[Bis(3-phenylthioureidomethyl)] benzene)
plasticizer (Fluka 73732)	91.6% wt	o-NPOE (o-nitrophenyl-n-octylether)
additive (Fluka 91661)	1.4% wt	TDDMACl (tridodecylmethylammonium chloride)
matrix PVC (Fluka 81392)	5% wt	NA
solvent (Sigma-Aldrich 83360)	Volumes relative to o-NPOE (2)	THF (tetrahydrofuran)

All chemicals were Selectophore^®^ grade.

**Table 2:** Average water characteristics in the membrane-aerated biofilm reactor (MABR).

Water Characteristics	Influent	Effluent
Dissolved Oxygen (DO) (mg/L)	8.6 ± 0.5	less than 0.85
Oxidation Reduction Potential (ORP) (mV)	350	−250~−350
Temperature (°C)	23	23
pH	7.6 ± 0.2	7.6 ± 0.2
SO_4_^2−^ (mg/L)	227 ± 21	90 ± 17
